# Psychosocial effects of a behavioural augmentation of existing public physical activity programs for middle-aged and older adults in Ireland

**DOI:** 10.1371/journal.pone.0318911

**Published:** 2025-03-04

**Authors:** Enrique García Bengoechea, Ciaran Doyle, Chloe Forte, Andrew O’Regan, Amanda M. Clifford, Stephen Gallagher, Alan Donnelly, Liam Glynn, Andrew W. Murphy, Ali Sheikhi, Catherine B. Woods

**Affiliations:** 1 Department of Physical Education and Sport Sciences, Physical Activity for Health Research Centre, Health Research Institute, University of Limerick, Ireland; 2 Centre for Public Health, Population Health Sciences, Bristol Medical School, University of Bristol,; 3 School of Medicine, Health Research Institute, University of Limerick, Ireland; 4 School of Allied Health, Health Research Institute, Ageing Research Centre, University of Limerick, Ireland; 5 Department of Psychology, University of Limerick, Ireland; 6 HRB Primary Care Clinical Trials Network, Discipline of General Practice, University of Galway,Ireland; 7 Health Research Institute, University of Limerick; Shahrood University of Medical Sciences, IRAN, ISLAMIC REPUBLIC OF

## Abstract

The combination of an ageing population, increasing prevalence of preventable noncommunicable diseases and a decline in physical activity with age emphasizes the need for investment in physical activity programs and services for older people. This study aimed to add to the initial evidence on the effectiveness of the Move for Life (MFL) intervention by examining its effects on psychosocial health outcomes and determinants of physical activity. MFL is an intervention that aims to augment existing community-based public physical activity programs for middle-aged and older adults in Ireland with strategies derived from behavioural theory and support from peer leaders. A 3-arm cluster randomised feasibility trial compared MFL intervention, usual provision (UP) and waiting list control (CON) groups at baseline (T0), post-intervention (T1, at 8-, 10- or 12-weeks) and 6-month follow up after baseline (T2). Psychosocial health and determinants of physical activity were assessed at each occasion by validated self-report measures. Linear or generalized linear mixed models were fitted to estimate group differences over time. Of 733 recruited individuals, 601 (mean age: 63.06 ± 8.1 years, 80.4% female) met study inclusion criteria. Significant advantages were found in the MFL group relative to UP in ratings of self-efficacy to overcome barriers to physical activity participation, subjective norms for and attitudes towards participation in physical activity (ps < .05). Subsequent analyses accounting for implementation fidelity revealed additional advantages for the ‘high fidelity’ MFL group relative to other groups, notably regarding loneliness and relatedness to others, perceived behavioural control, attitudes toward and intentions to participate in physical activity (ps < .05). The pattern of results shows the potential of MFL to impact positively the psychosocial health of inactive adults aged 50 + years and change psychosocial determinants of physical activity, particularly when implemented as intended. The results suggest as well that existing physical activity programs may have unexpected psychosocial consequences.

## Introduction

As life expectancy around the world increases, the importance of healthy ageing and preventing avoidable falls and injuries becomes an increasingly important health and economic issue [1]. Adults not meeting recommended physical activity (PA) guidelines, regardless of their current health status, are a key target for intervention as they face potential future risk of developing ill health without long-term lifestyle change [2]. Worldwide, insufficient PA  is a major modifiable risk factor for chronic illness and premature mortality [3]. Globally, noncommunicable diseases (NCDs) pose significant costs to population health and to the economy [4]; yet are largely preventable. Research indicates that ageing is associated with more chronic illnesses [5], and reduced participation in PA [6]. In Ireland, 35% of adults aged 55-65 years and 18% aged > 75 years reported achieving recommended minimum PA levels for health [7].

The combination of an ageing population, growing prevalence of NCDs that can be prevented addressing modifiable risk factors and a decline in PA with age underscores the need for investment in PA programs and services for older people [1]. To influence population health, interventions must be scaled up in real-world contexts [8]. There is a need for effective older adult PA interventions that are planned with maintenance and scale-up in mind, that consider implementation evaluation a priori, and interpret health impact within the context of implementation factors [9]. In particular, there are very few PA interventions that include assessment of implementation fidelity (i.e., extent to which an intervention is delivered as planned or intended) and seek to relate quality of implementation of an intervention to the health impact for participants [2,9]. Furthermore, there is a need for PA programmes for older adults targeting the least active within this demographic group and with a focus on maintenance of PA [10]. Few older adult intervention studies are guided by implementation or scale-up frameworks [9]. Guided by an Intervention Mapping planning framework [11], Move for Life (MFL) was developed to enable inactive adults aged 50 + years to meet PA guidelines [12,13]. MFL drew on both traditional evidence-to-practice and complementary practice-to-evidence pathways for its development [8,14], and was designed with sustainability and scalability considerations in mind [15]. Anchoring the programme within the existing public Local Sport Partnership (LSP) network in Ireland ensured it was embedded in and would benefit from a well-established community organisation and structure. The MFL intervention is an ‘augmentation’ aiming to enrich existing LSP programmes, instead of a new programme [12], which was key to improving the prospect of adoption within and scale-up across the network of LSPs. MFL consists of three components designed to target theory derived behaviour change techniques deemed as the active ingredients for change: a training workshop for LSP professional PA instructors supported by a programme handbook, a training workshop for peer mentors, and a programme handbook for MFL participants. Initial evidence provides support for its sustained positive impact on energy expenditure-related outcomes, body composition, physical function and wellbeing over a period of six months [16].

Beyond changes related to physical health, changes in the domain of mental health and wellbeing may be expected as well as a result of well-planned PA interventions [17,18]. In fact, in some cases these may be the only changes observed following PA interventions [19]. Given the importance of mental health for overall health and wellbeing, research should focus on examining the effects of PA interventions on mental health [20]. Despite the modest but growing body of evidence for interventions designed to increase PA in older adults, there is a need for studies addressing neglected outcomes (e.g., those related to psychosocial health and wellbeing), populations and settings [21]. From the point of view of the hypothesized mechanisms through which MFL affects behaviour change, there is also a need to examine the effects of the intervention on psychosocial mediators or determinants of change supported by theory [12]. Consequently, the aim of this study was to extend the existing evidence of effectiveness for  the MFL intervention by examining the effects of the intervention on psychosocial outcomes and theory-based determinants of PA in a sample of inactive adults aged 50 years and over from predominantly socioeconomically disadvantaged areas in Ireland.

## Methods

### Setting and participants

Eight LSP ‘sport and PA community hubs’ in mid-west Ireland were recruited. Hub inclusion criteria required professional expertise to run four nationally approved PA programmes suitable for inactive older adults. These were *Men on the Move* (an evidence-based mixed sport programme for men; 12 weeks, 2 sessions/week [22])*, Women on Wheels/Bike for Life* (a ‘Get Ireland Cycling’ cycling programme; 10 weeks, 1 session/week)*, Go for Life* (an ‘Age & Opportunity’ indoor mixed games programme; 8 weeks, 1 session/week) *and Get Ireland Walking* (an outdoor community walking programme; 10 weeks, 1 session/week). In total, 32 PA programmes were run over the trial period. MFL recruited 733 individuals (May-September, 2018).

Recruitment strategies, informed by previous qualitative research [13], included radio interviews, press releases in national and local papers, notices in newsletters and community notes, notices read out at religious services, presentations to local groups, presentations at General Practitioners meetings, notices to library groups, text messages via community alerts, posters and leaflet drops at shops, resource centres, bingo groups, etc., and social media posts via various targeted interest groups. To be included in the trial, participants had to be physically inactive, community dwelling, aged 50 years plus during the year the intervention took place, and able to exercise independently.

### Study design

A cluster design was used to overcome potential for contamination in the form of spillover effects (e.g., exposure to intervention behaviour change techniques) that could arise if participants assigned to different trials arms were not geographically separated. To this end, eight LSP hubs in Limerick and Clare were assigned as the units of randomisation (the clusters). Participants within these hubs (units of analysis) were randomised to one of three arms, i) the MFL intervention group (MFL; the PA programme plus the MFL augmentation, 3 hubs); ii) usual provision (UP; the PA programme delivered as usual, 3 hubs); and iii) the waiting list control group (CON; information on PA only, 2 hubs). CON participants received an invitation to participate in the PA programmes once the trial was completed. Each hub was geographically separated to reduce potential for spillover effects and clusters were stratified as rural or urban. Randomisation of hubs occurred following baseline assessment and was conducted by a researcher external to the study team using a process of minimisation [23]. The participants allocated to the MFL intervention group and the usual PA programmes were not aware of their status. Detailed information on the MFL trial protocol is available elsewhere [24].

### Intervention

The MFL intervention is described in detail elsewhere [12]. In brief, MFL aimed to enhance the impact of established national PA programmes by augmenting the professional model (LSP PA instructors) with training in behavioural theory: social cognitive theory [25], self-determination theory [26], and group dynamics concepts focusing on integration and cohesion [27]. Additionally, the intervention sought to identify and recruit suitable peer mentors among the participants in the PA programmes who were subsequently trained by researchers on how to sustain their PA group in the long-term. Specifically, a three-hour training workshop was developed and delivered for the peer mentors by the research team. The training programme covered the rationale for mentoring and its potential impact on the PA behaviour of group members, principles of effective communication with mentees derived from motivational interviewing, suggestions to support the role of the LSP PA instructors, and ideas to keep the group together beyond the end of the regular PA programmes.

MFL handbooks supported the training with a protocol for the delivery type, frequency, and intervention content [12]. Table 1 shows the intervention strategies and content covered in the training for PA instructors delivering the intervention. As shown in Table 1, intervention strategies had three main aims: 1) help participants develop cognitive and behavioural skills (e.g., goal setting andself-monitoring) to regulate their own PA behaviour, 2) offer opportunities to socialise, give and receive support, and develop feelings of connectedness and belonging, and 3) build group integration and cohesion by fostering positive group dynamics and developing a sense of group identity around norms for participation in PA. Training was tailored to meet group and individual needs and supported by a MFL researcher who assisted instructors and peer mentors throughout the study period. The PA programmes and intervention took place from 2018-2019.

**Table 1:** Content delivered in MFL training workshop for LSP instructors and behaviour change strategies used in the MFL intervention (adapted from Bengoechea et al., 2021).

Cognitive and behavioural skills (social cognitive theory)	
Content delivered	MFL strategy
Reasons for participation Benefits of PA Knowledge of PA guidelines Rating perceived effort Opportunities to increase PA Decisional balance (pros and cons) Goal setting and self-monitoring Overcoming barriers/problem solving Relapse prevention	Instructor-facilitated group discussion (e.g., provide information, reinforce progress, address barriers) Partnered and small group activities involving sharing/discussion ‘Homework’ weekly handouts (concise info + self-reflection)
**Social support (self-determination theory)**	
**Content delivered**	**MFL strategy**
Building, strengthening, and maintaining social networks that support increases in PA Instrumental (providing direct assistance) Informational (sharing knowledge about resources) Emotional (demonstrating concern, caring or affection)	Partnered and small group activities involving sharing/discussion Ongoing feedback and encouragement from other participants PA contract Buddy system ‘Social time’ after activity
**Group dynamics (group integration and cohesion concepts)**	
**Content delivered**	**MFL strategy**
Providing opportunities for group social interaction Fostering positive group dynamics Developing appropriate group norms Developing sense of group identity/distinctiveness Building a sense of group integration around task and social aspects of activity	Cooperative activities and group challenges ‘Friendly’ competition games Develop sense of group distinctiveness Assign group roles to participants Group goal setting and problem solving ‘Social time’ after activity Occasional ‘get togethers’ outside of activity

MFL =  Move for Life, LSP =  Local Sports Partnership, PA =  physical activity.

### Procedure

All experimental protocols were approved by the University of Limerick, Faculty of Education and Health Sciences Research Ethics Committee (Registration No. 2018_02_15_EHS; 09 April 2018) and were carried out in accordance with the Declaration of Helsinki for research involving human subjects. Individuals were informed about the study in person, and in writing. A diverse range of recruitment strategies informed by qualitative research with stakeholders were used. This research, published elsewhere [13], required separate ethical approval (Registration No. 2018_01_04_EHS; 06 March 2018). Individuals who expressed an interest in the MFL trial attended a ‘health check appointment’ where they received further information about this study in person, and in writing. Recruitment of participants for the MFL trial took place from 01/05/2018 to 30/05/2018 (Limerick) and from 01/08/2018 to 28/09/2018 (Clare). All participants provided written informed consent. Confidentiality was ensured trough strict data handling and storage, in accordance with the Research Code of Ethics and Data Protection Guidelines. Since the data was not identifiable by individual participants, preassigned individualised identification codes allowed tracking of participants and facilitate longitudinal analyses. An Adverse Events Reporting System was put in place, reporting back to the MFL advisory committee for decisions about trial safety and continuance [24].

Consenting individuals completed baseline measures and their hubs were subsequently assigned to the CON, UP or MFL arm. Study measures were collected at baseline (T0), post-intervention (T1, at 8-, 10- or 12-weeks), and 6-month follow up after baseline (T2). Timing of post-intervention measures varied because of the different duration of the LSP PA programmes included the MFL trial, which has been noted earlier.

### Measures

Questionnaires collected data on demographics (age, gender, marital status, education level, health insurance and occupational status). Additionally, several measures of psychosocial health and determinants of PA, which have shown appropriate psychometric properties in previous research using adult samples, were used as study outcomes. Perceived relatedness in physical activity contexts was assessed using the 6 item Relatedness to Others in Physical Activity Scale (ROPAS) [28]. Emotional loneliness was assessed using a modified version of the UCLA Loneliness Scale [29]. The five item Exercise Self Efficacy Scale (ESE) [30] was used to measure participants perceptions about their abilities to engage in exercise under different situations. The Decisional Balance Scale was used to assess participants perceptions about the positives and negatives associated with PA participation [31]. Participants were asked questions related to Theory of Planned Behaviour (TPB) [32] variables with reference to completing 30 minutes of PA per day. TPB variables assessed were attitude to PA, perceived behavioural control, subjective norms, and intention. Each of the TPB variables have been used in previous studies [33–35] and have demonstrated acceptable internal consistencies. Lastly, to assess self-rated health, participants were also asked to complete the ‘feeling thermometer’ on the EuroQuol-5 Dimension-5 Level (EQ-5D-5L) measure [36].

Fidelity to prescribed MFL intervention content was assessed weekly by instructor fidelity checklists [37] monitored by a MFL researcher with phone calls. Based on aggregates of weekly checklist data, average compliance with intervention strategies was calculated.

### Data analysis

Descriptive statistics were summarised by trial arm at baseline. Following initial diagnosis of distributional and missing data (missing at random) assumptions, linear mixed models, or generalised linear mixed models with robust estimation, were used to calculate the adjusted differences in means of study outcomes between groups post-intervention and at follow up, and explore differences in change over time. Observations were nested within participants to account for the hierarchical structure of data and autocorrelation due to repeated measures.

Guided by an ecological perspective of active living [38], each of several potential covariates of the outcomes considered (S1 Table) were examined to understand how they relate, on their own, to the initial status and rate of change of the outcomes. In addition, LSP, by which the randomisation was stratified, was accommodated by its inclusion as covariate. Likewise, a categorical variable ‘Group’ was examined as covariate to explore any trial arm differences in the initial status and change over time in a linear (i.e., interaction with time) or nonlinear/quadratic (i.e., interaction with time squared) fashion.

For each outcome, variables with p-values >  0.1 in the preliminary models examining bivariate associations, and variables central to the research questions (e.g., Group, and its interaction with Time, LSP), were included in a subsequent multivariate model. Several covariance structures appropriate for longitudinal data were tested to determine the error covariance structure that best fit the data.

Analyses followed an intention-to-treat principle and all available observations were used to estimate the models. Differences in adjusted means at each measurement point and Group x Time interaction coefficients are presented with their corresponding 95% confidence intervals and p-values. Statistical significance was set at p <  0.05. All analyses were conducted using Statistical Product and Service Solutions (SPSS) version 27.

The initial analysis comparing the three intervention groups (MFL, UP, CON) was complemented with an analysis in which the MFL group was split into a ‘high fidelity’ group (highest tertile) and a ‘medium and low fidelity’ group (remaining tertiles combined), based on available fidelity to intervention content scores, and compared to each other and the remaining groups.

## Results

Out of 733 recruited individuals, 98% (n = 724) consented and completed baseline measures. Of those 18% (n = 132) did not meet the eligibility criteria and were excluded due to age (less than 50 years) or activity status (meet PA guidelines). Due to the feasibility nature of the trial no formal sample size calculations were conducted [16]. Excluded individuals were younger (59.4 vs 63.06, p < .001), more active (activPAL MVPA mins (10 minutes bouts) 32.12 vs 13.02, p < .001) and predominantly male (29.9% vs 19.6%, p < .01). Table 2 presents the baseline demographic characteristics of 601 included participants. Most were female (80.4%), 37% were living with > 3 chronic conditions, and 41% were obese. Trial arms were well balanced at baseline, with age and marital status the only significant differences between arms. UP were older than other participants, and CON were more likely to be separated or divorced than those in the other arms. CON participants also presented with a more favourable body composition profile in terms of body mass index (BMI) and waist circumference of all three groups.

**Table 2:** Baseline (T0) characteristics of study participants by trial arm (n = 601).

Descriptive Variables	Study Group		
	MFL	UP	CON
Age: mean (SD), N	61.86 (7.98), 189	64.22 (8.51), 269	62.50 (7.37), 143
Gender: N (%)			
Male	29 (15.3)	58 (21.6)	31 (21.7)
Female	160 (84.7)	211 (78.4)	112 (78.3)
Level of Education: N (%)			
Primary or no formal training	16 (8.7)	22 (8.4)	6 (4.3)
Lower secondary	30 (16.3)	37 (14.1)	23 (16.3)
Upper secondary	50 (27.2)	57 (21.8)	32 (22.7)
Post-secondary, non-tertiary	6 (3.3)	17 (6.5)	3 (2.1)
Non degree	35 (19.0)	65 (24.8)	44 (31.2)
Degree or higher	47 (25.5)	64 (24.4)	33 (23.4)
Medical Card: N (%)			
Yes	56 (30.8)	102 (38.9)	45 (31.9)
No	126 (69.2)	160 (61.1)	96 (68.1)
Marital Status: N (%)			
Married/living with partner	128 (69.2)	170 (64.6)	87 (61.7)
Other	57 (30.8)	93 (35.4)	54 (38.3)
Body mass index: mean (SD), N	30.19 (6.08), 188	29.37 (5.44), 261	28.91 (5.50), 142
Waist circumference: mean (SD), N	98.88 (15.8), 189	96.23 (13.9), 263	94.78 (14.2), 141
Number of chronic health conditions: mean (SD), N	2.53 (1.44), 150	2.77 (1.63), 204	2.47 (1.33), 101
Area deprivation index: N (%)			
Marginally below average	189, (100.0)	180 (66.9)	49 (43.3)
Disadvantaged	–	89 (33.1)	94 (65.7)
Geographical Location: N (%)			
Rural	189 (100.0)	53 (19.7)	–
Urban	–	216 (80.3)	143 (100.0)

MFL = Move for Life, UP= Usual Provision, CON = Control.

Fidelity with MFL strategies was 77% (508 out of required 662 intervention strategies delivered as prescribed). The study retention rate was 63%, with MFL, UP, and CON groups achieving retention rates of 64%, 58% and 79% respectively. Missing observations for participants included in the analyses ranged from 23% to 27%. Missing observations were handled using maximum likelihood estimation in the linear or generalized linear mixed regression models to use all available data to estimate the parameters of interest. This method has been shown to be a robust approach that can be provide unbiased estimates under the assumption of missing at random [39], which was tenable in our data.

The unadjusted means of the primary and secondary outcomes and number of participants at each time point are shown in S2 Table, while Table 3 displays the percentage change in unadjusted means of the study outcomes for each group. Tables 4 and 5 show differences in adjusted means between groups at T0, T1 and T2 with corresponding p-values. In addition, the tables show ‘Group x Time’ interaction coefficients, and their p-values, examining whether changes in study outcomes over time (T0, T1, T2) vary as a function of treatment condition using original groups (MFL, UP, CON) or groups split according to fidelity scores (MFL high fidelity, MFL medium and low fidelity, UP, CON), respectively.

**Table 3:** Percentage change in outcome variables according to study group and period.

	T0-T1			T0-T2		
	MFL	UP	CON	MFL	UP	CON
Health T	7.08%	8.44%	6.46%	7.15%	6.43%	3.01%
Loneliness	0%	-0.67%	-4.70%	-4.70%	-2.68%	-2.68%
Relatedness	6.62%	-2.18%	-1.53%	3.75%	-1.74%	-3.06%
Dec bal	-3.91%	-5.1%	0.51%	-6.15%	-3.57%	-8.72%
Barriers SE	5.58%	-6.64%	1.03%	4.83%	-4.9%	2.06%
Attitude	-2.07%	-3.56%	0.60%	-2.07%	-2.97%	1.51%
Subj norm	0%	-2.52%	5.38%	0.35%	-1.44%	5%
Beh control	-3.28%	-2.12%	-2.39%	-4.78%	-2.73%	-0.90%
Intention	-4.19%	-5.78%	-3.11%	-6.29%	-7.6%	-1.24%

MFL =  Move for Life, UP = Usual Provision, CON =  Control. Healt T =  health thermometer (self-rated health); Dec bal =  decisional balance; Barriers SE =  barriers self-efficacy; Subj norm =  subjective norms; Beh control =  perceived behavioural control. Percentage changes are based on unadjusted means.

**Table 4:** Group differences over time in psychosocial outcome variables.

	Adjusted mean difference (95% CI) T0	p-value	Adjusted mean difference (95% CI) T1	p-value	Adjusted mean difference (95% CI) T2	p-value	Group x Time interaction^a^ (95% CI)	p-value
**Health T**								
MFL vs CON	−3.73 (−7.39, −0.14)	.042	−2.96 (−6.27, 0.35)	.080	−2.54 (−5.68, 0.61)	.114	1.43 (−0.88, 3.74)	.225
MFL vs UP	0.42 (−2.72, 3.55)	.794	0.32 (−2.50, 3.15)	.822	0.17 (−2.61, 2.95)	.903	−0.30 (−2.50, 1.91)	.793
UP vs CON	−4.14 (−7.74, −0.55)	.024	−3.28 (−6.51, −0.06)	.046	−2.71 (−5.72, 0.31)	.078	1.73 (−0.58, 4.03)	.141
**Loneliness**								
MFL vs CON	0.06 (−0.04, 0.15)	.246	0.07 (−0.02, 0.16)	.110	0.06 (−0.03, 0.15)	.182	0.01 (−0.04, 0.05)	.823
MFL vs UP	0.01 (−0.08, 0.09)	.862	−0.001(−0.08, 0.08)	.977	−.017 (−0.10, 0.06)	.666	−0.03 (−0.07, 0.02)	.192
UP vs CON	0.05 (−0.04, 0.13)	.270	0.08 (−0.01, 0.16)	.073	0.08 (−0.003, 0.16)	.058	0.03 (−0.01, 0.08)	.117
**Relatedness**								
MFL vs CON	0.28 (−0.17, 0.74)	.225	0.22 (−0.18, 0.62)	.288	0.18 (−0.18, 0.54)	.334	−0.12 (−0.41, 0.16)	.400
MFL vs UP	0.26 (−0.13, 0.64)	.189	0.23 (−0.11, 0.57)	.179	0.20 (−0.09, 0.50)	.180	−0.06 (−0.29, 0.16)	.584
UP vs CON	0.03 (−0.23, 0.28)	.848	−0.01 (−0.24, 0.21)	.903	−.026 (−0.23, 0.18)	.808	−0.06 (−0.22, 0.11)	.489
**Dec bal**								
MFL vs CON	−0.16 (−0.37, 0.05)	.133	−0.17 (−0.36, 0.02)	.073	−0.16 (−0.33, 0.02)	.080	0.004 (−0.14, 0.14)	.960
MFL vs UP	−0.15 (−0.34, 0.03)	.092	−0.17 (−0.33, −0.01)	.043	−0.20 (−0.36, −0.05)	.010	−0.06 (−0.18, 0.07)	.376
UP vs CON	−0.01 (−0.20, 0.19)	.962	−0.002 (−0.18, 0.18)	.980	0.05 (−0.12, 0.21)	.587	0.06 (−0.07, 0.19)	.377
**Barriers SE**								
MFL vs CON	−0.09 (−0.28, 0.11)	.390	−0.08 (−0.26, 0.10)	.380	−0.08 (−0.25, 0.10)	.377	0.01 (−0.10, 0.12)	.872
MFL vs UP	−0.03 (−0.20, 0.13)	.686	0.03 (−0.13, 0.18)	.722	0.06 (−0.09, 0.20)	.441	0.11 (0.01, 0.21)	.033^†^
UP vs CON	−0.05 (−0.23, 0.13)	.582	−0.11 (−0.28, 0.06)	.207	−0.14 (−0.30, 0.03)	.101	−0.10 (−0.21, 0.01)	.073
**Attitude**								
MFL vs CON	0.03 (−0.06, 0.12)	.506	−0.04 (−0.12, 0.04)	.302	0.02 (−0.05, 0.10)	.544	−0.05 (−0.11, 0.02)	.139
MFL vs UP	−0.04 (−0.12, .04)	.326	−0.004 (−0.09, 0.08)	.927	0.04 (−0.05, 0.12)	.381	0.03 (−0.03, 0.09)	.290
UP vs CON	0.07 (−0.01, 0.16)	.095	−0.04 (−0.11, 0.04)	.323	−0.01 (−0.08, 0.06)	.716	−0.08 (−0.14, −0.02)	.005^†^
**Subj norm**								
MFL vs CON	0.32 (0.14, 0.50)	.001	0.31 (0.12, 0.50)	.001	0.28 (0.11, 0.46)	.001	−0.04 (−0.11, 0.03)	.228
MFL vs UP	0.18 (0.02, 0.33)	.023	0.22 (0.06, 0.38)	.006	0.21 (0.07, 0.36)	.004	0.04 (−0.02, 0.11)	.178
UP vs CON	0.15 (0.03, 0.26)	.012	0.09 (−0.02, 0.20)	.091	0.07 (−0.03, 0.17)	.168	−0.09 (−0.15, −0.02)	.011^†^
**Beh control**								
MFL vs CON	−0.01 (−0.18, 0.17)	.949	0.02 (−0.14, 0.18)	.839	0.03 (−0.12, 0.18)	.659	0.05 (−0.06, 0.15)	.398
MFL vs UP	−0.01 (−0.15, 0.14)	.951	0.03 (−0.11, 0.16)	.711	0.07 (−0.06, 0.19)	.296	0.09 (−0.003, 0.17)	.059
UP vs CON	−0.001 (−0.10, 0.10)	.984	−0.01 (−0.10, 0.08)	.846	−0.03 (−0.12, 0.06)	.462	−0.04 (−0.11, 0.03)	.258
**Intention**								
MFL vs CON	0.11 (0.002, 0.22)	.046	0.09 (−0.01, 0.18)	.083	0.05 (−0.04, 0.14)	.292	−0.08 (−0.15, −0.002)	.045^†^
MFL vs UP	0.02 (−0.08, 0.11)	.760	0.03 (−0.06, 0.12)	.454	0.05 (−0.03, 0.14)	.217	0.05 (−0.02, 0.11)	.185
UP vs CON	0.10 (−0.003, 0.20)	.058	0.05 (−0.04, 0.15)	.269	−0.004 (−0.09, 0.08)	.924	−0.12 (−0.20, −0.05)	.001^†^

MFL = Move for Life Intervention Group, UP = Usual Provision Group, CON = Control Group; Healt T = health thermometer (self-rated health); Dec bal = decisional balance; Barriers SE = barriers self-efficacy; Subj norm = Subjective norms; Beh control = perceived behavioural control.

^†^Significant ‘Group x Time’ interaction indicative of intervention effects. Models are adjusted by Local Sports Partnership and relevant demographic, biological, social, and environmental variables.

**Table 5:** Group differences over time in psychosocial outcome variables considering fidelity to intervention content.

	Adjusted mean difference (95% CI) T0	p-value	Adjusted mean difference (95% CI) T1	p-value	Adjusted mean difference (95% CI) T2	p-value	Group x Time interaction (95% CI)	p-value
**Health T**								
MFL high vs CON	−4.38 (−10.17, 1.40)	0.137	−4.19 (−9.56, 1.17)	0.125	−3.72 (−8.87, 1.43)	0.156	0.78 (−1.91, 3.47)	0.572
MFL medium-low vs CON	−3.58 (−7.61, 0.45)	0.081	−2.46 (−6.13, 1.22)	0.189	−1.78 (−5.33, 1.77)	0.325	2.12 (−0.69, 4.93)	0.139
UP vs CON	−4.18 (−7.77, −0.58)	0.023	−3.31 (−6.53, −.08)	0.044	−2.72 (−5.73, 0.30)	0.077	1.72 (−0.58, 4.02)	0.143
**Loneliness**								
MFL high vs CON	0.07 (−0.07, 0.20)	0.36	0.07 (−0.06, 0.21)	0.271	0.04 (−0.09, 0.17)	0.548	−0.03 (−0.09, 0.03)	0.341
MFL medium-low vs CON	0.08 (−0.04, 0.19)	0.211	0.10 (−0.02, 0.21)	0.105	0.09 (−0.03, 0.20)	0.126	0.02 (−0.04, 0.07)	0.594
UP vs CON	0.05 (−0.04, 0.13)	0.264	0.08 (−0.01, 0.16)	0.066	0.08 (−0.001, 0.16)	0.054	0.03 (−0.01, 0.08)	0.114
MFL high vs UP	0.01 (−0.12, 0.14)	0.906	−0.002 (−0.13, 0.12)	0.975	−0.04 (−0.16, 0.08)	0.53	−0.06 (−0.13, −0.003)	.041†
**Relatedness**								
MFL high vs CON	0.62 (0.11, 1.13)	0.018	0.51 (0.07, 0.94)	0.022	0.44 (0.05, 0.82)	0.027	−0.21 (−0.55, 0.14)	0.239
MFL medium-low vs CON	0.17 (−0.33, 0.67)	0.502	0.09 (−0.35, 0.53)	0.682	0.06 (−0.34, 0.46)	0.768	−0.13 (−.042, 0.17)	0.404
UP vs CON	0.01 (−0.24, 0.27)	0.913	−0.03 (−0.25, 0.20)	0.82	−0.04 (−0.25, 0.17)	0.73	−0.06 (−0.22, 0.11)	0.495
**Dec bal**								
MFL high vs CON	−0.11 (−0.43, 0.20)	0.481	−0.12 (−0.40, 0.16)	0.408	−0.07 (−0.34, 0.19)	0.577	0.05 (−0.16, 0.25)	0.655
MFL medium-low vs CON	−0.18 (−0.42, 0.07)	0.158	−0.17 (−0.40, 0.05)	0.123	−0.14 (−0.34, 0.07)	0.191	0.05 (−0.11, 0.21)	0.565
UP vs CON	−0.01 (−0.20, 0.19)	0.942	−0.01 (−0.18, 0.17)	0.958	0.04 (−0.12, 0.21)	0.597	0.06 (−0.07, 0.19)	0.379
**Barriers SE**								
MFL high vs CON	−0.19 (−0.49, 0.10)	0.193	−0.15 (−0.42, 0.12)	0.282	−0.11 (−0.37, 0.15)	0.404	0.10 (−0.06, 0.26)	0.234
MFL medium-low vs CON	−0.10 (−0.32, 0.13)	0.395	−0.10 (−0.31, 0.11)	0.352	−0.11 (−0.31, 0.09)	0.263	−0.02 (−0.15, 0.11)	0.78
UP vs CON	−0.05 (−0.23, 0.13)	0.593	−0.11 (−0.28, 0.06)	0.201	−0.14 (−0.30, 0.03)	0.103	−0.10 (−0.21, 0.01)	0.073
**Attitude**								
MFL high vs CON	0.04 (−0.10, 0.17)	0.59	−0.04 (−0.12, 0.03)	0.276	0.02 (−0.06, 0.09)	0.695	0.03 (−0.07, 0.12)	0.578
MFL medium-low vs CON	0.02 (−0.09, 0.13)	0.698	0.02 (−0.09, 0.13)	0.723	0.05 (−0.05, 0.16)	0.308	−0.09 (−0.16, −0.01)	.033†
UP vs CON	0.08 (−0.01, 0.16)	0.079	−0.08 (−0.16, 0.02)	0.101	−0.06 (−0.14, 0.03)	0.206	−0.08 (−0.14, −0.02)	.006†
MFL high vs UP	0.01 (−0.11, 0.12)	0.935	0.03 (−0.07, 0.13)	0.519	0.07 (−0.02, 0.16)	0.145	0.11 (0.01, 0.20)	.013†
**Subj norm**								
MFL high vs CON	0.30 (0.07, 0.53)	0.01	0.32 (0.10, 0.55)	0.006	0.33 (0.12, 0.54)	0.002	0.03 (−0.07, 0.14)	0.521
MFL medium-low vs CON	0.27 (0.08, 0.47)	0.007	0.27 (0.06, 0.47)	0.01	0.23 (0.04, 0.41)	0.016	−0.05 (−0.14, 0.03)	0.201
UP vs CON	0.14 (0.03, 0.26)	0.012	0.09 (−0.02, 0.20)	0.098	0.07 (−0.03, 0.17)	0.173	−0.08 (−0.15, −0.02)	.013†
**Beh control**								
MFL high vs CON	−0.01 (−0.22, 0.20)	0.941	0.05 (−0.15, 0.24)	0.646	0.08 (−0.10, 0.25)	0.38	0.10 (−0.03, 0.23)	0.13
MFL medium-low vs CON	−0.11 (−0.30, 0.08)	0.274	−0.09 (−0.27, 0.08)	0.299	−0.08 (−0.24, 0.09)	0.356	0.03 (−0.09, 0.16)	0.577
UP vs CON	0.01 (−0.09, 0.11)	0.905	−0.003 (−0.10, 0.09)	0.956	−0.03 (−0.12, 0.06)	0.537	−0.04 (−0.11, 0.03)	0.243
MFL high vs UP	−0.03 (−0.23, 0.18)	0.807	0.05 (−0.12, 0.22)	0.586	0.11 (−0.05, 0.26)	0.178	0.14 (0.03, 0.26)	.016†
**Intention**								
MFL high vs CON	0.14 (−0.04, 0.32)	0.125	0.13 (−0.03, 0.29)	0.115	0.11 (−0.05, 0.26)	0.174	−0.04 (−0.16, 0.07)	0.451
MFL medium-low vs CON	0.05 (−0.09, 0.18)	0.483	0.03 (−0.09, 0.14)	0.644	−0.01 (−0.12, 0.10)	0.796	−0.07 (−0.16, 0.01)	0.096
UP vs CON	0.10 (0.002, 0.20)	0.046	0.06 (−0.04, 0.15)	0.235	−0.001 (−0.09, 0.09)	0.991	−0.12 (−0.19, −0.04)	.002†

MFL = Move for Life Intervention Group, UP = Usual Provision Group, CON = Control Group; Healt T = health thermometer (self-rated health); Dec bal = decisional balance; Barriers SE = barriers self-efficacy; Subj norm = Subjective norms; Beh control.

### Initial analysis comparing intervention groups

As seen in Table 4, we found a significant Group x Time interaction for subjective norms, which declined over time in UP relative to CON (B =  -0.09; 95% C.I. =  -0.15, -0.02) and also in absolute terms (see percentage changes in Table 3). Consistent with this pattern, attitude towards PA became significantly less favourable over time among UP participants when compared to CON participants (B =  -0.08; 95% C.I. =  -0.14, -0.02) and in absolute terms as well. In addition, self-efficacy to overcome PA barriers increased in the MFL group relative to the UP group (B =  0.11; 95% C.I. =  0.01, 0.21). Specifically, self-efficacy increased over time in the former and decreased in the latter (Tables 3 and 4). Lastly, as the significant Group x Time interaction coefficients in table 4 indicate, intention to participate in PA decreased significantly in both the MFL group (B =  -0.08; 95% C.I. =  -0.15, -0.002) and, particularly, the UP group (B =  -0.12; 95% C.I. =  -0.20, -0.05) when compared to the CON group. As the percentage changes in Table 3 show, intention to participate in PA decreased in the three groups from T0-T2, although the decrease was smaller among CON participants.

### Analysis considering level of implementation fidelity

While the pattern of findings in the secondary analysis was generally consistent with the pattern observed in the main analysis, several differences emerged (Table 5). Notably, levels of loneliness among ‘high fidelity’ MFL participants decreased over time when compared to participants in the UP group (B =  -0.06; 95% C.I. =  -0.13, -0.003) and also in absolute terms. Furthermore, although ratings in perceived behavioural control among participants in the ‘high fidelity’ MFL group decreased slightly from T0-T2, they decreased significantly less than among UP participants (B =  0.14; 95% C.I. =  0.03, 0.26). The ‘high fidelity’ MFL group was also the only group in which attitude towards PA did not become significantly less favourable over time compared to the CON group (B =  0.03; 95% C.I. =  -0.07, 0.12). In fact, as indicated by the corresponding interaction coefficient in Table 5, attitudes among participants in this group worsened significantly less over time than among UP participants (B =  0.11; 95% C.I. =  0.01, 0.20). Similarly, relative to the CON group, intention to participate in PA declined significantly in the UP group (B =  -0.12; 95% C.I. =  -0.19, -0.04). Although intention to participate declined as well over time among participants in both MFL groups, such decline was not significant when compared to each other or CON (Tables 3 and 5). Lastly, even though the coefficient of interaction between group and time did not reach the specified level of statistical significance, unlike the initial analysis (Table 4), the difference in adjusted means of relatedness to others between the ‘high fidelity’ MFL group and CON became significant, and favourable to the former, both at post-intervention (T1) (mean difference =  0.51; 95% C.I. =  0.07, 0.94) and follow-up (T2) (mean difference =  0.44; 95% C.I. =  0.05, 0.82) (Table 5).

## Discussion

This study examined the effects of the MFL cluster randomised trial on psychosocial outcomes and theory-based determinants of PA. When considering the analysis using the three intervention groups (MFL, UP, CON), the psychosocial benefits of the MFL augmentation compared to the regular PA programs were particularly evident in the circumstance that while self-efficacy to overcome barriers to PA increased in the MFL group over the duration of the study it decreased in the UP group. In addition, subjective norm ratings decreased over time in the UP group compared to the CON group, but not in the MFL group. Similarly, attitudes towards PA became less favourable over time only among UP participants when compared to CON participants. These findings are suggestive of a protective effect of the MFL intervention against patterns of response observed in participants from existing programs that may be indicative of unintended consequences of such programs.

In line with our findings concerning existing programs, previous research has found that perceived pressure to keep up with the group to complete program activity routines can lead to feelings of incompetence and disconnection from others; this is particularly true in group-based physical activities that involve people of different ages, gender, and physical capabilities, which was the case in our study [40]. Participation in PA programs can also exacerbate negative self-perceptions, especially in individuals who compare themselves unfavourably to others in the group [41]. Furthermore, pain and discomfort, as well as concerns with falling, which may all result from activities that are too intense or demanding, are major barriers to older adults engagement with PA [40]. While PA is generally associated with numerous benefits in older adults [42], some individuals may experience feelings of loneliness or isolation if they do not feel integrated into the group or if the activities do not foster meaningful social connections [43].

The differences in self-efficacy, subjective norms and attitudes favouring MFL over UP participants in particular can be explained by the use of cognitive and behavioural skills and social support and group cohesion intervention strategies derived from social cognitive theory [25], self-determination theory [26] and conceptual and intervention work in group integration/cohesion [27]. These aimed, for example, to help participants problem solve to identify and overcome common barriers to participation in PA, receive and give social support and develop appropriate group norms for participation [12]. In line with these findings, another study, assessing the effects of using peer volunteering support to promote active ageing in socially disengaged, inactive older adults in the UK [44], found that participants randomized to one-to-one support from an activator (intervention) reported increased confidence to get out and about, confidence in the face of specific barriers, and perceived social support compared to a waiting list control group post-intervention (6 months). Collectively, the findings from the two studies add to the evidence regarding the potential of peer support intervention strategies to increase PA participation among middle-aged and older adults.

While we expected a priori to see more differences between participants in the MFL and the CON groups, several circumstances might help explain the relative good performance of the CON group, particularly on indicators such as intention to participate in PA and when compared to the UP group. The CON group was a waiting list group made by participants who were not initially randomised to either the MFL or UP groups. As a result, we can speculate that CON participants were eagerly anticipating the end of the study period to have their request to register in one of the regular PA programs offered by their local LSP accommodated. Despite randomisation, as seen in Table 1, CON participants had the more favourable body composition profile in terms of BMI and waist circumference of all participants. The documented association between indicators of body composition and indicators of health and health-related quality of life in older community-dwelling adults [45] may in part explain the relative good standing of the CON group in terms of self-rated health, particularly when compared to UP participants, as per the differences in adjusted means at each time point.

Crucial to understanding the psychosocial effects of the MFL intervention augmentation, the analysis accounting for implementation fidelity revealed additional, and notable, benefits of the augmentation when delivered as intended. For example, even though the interaction did not reach statistical significance, the difference in adjusted means of relatedness between the ‘high fidelity’ MFL group and CON was significant, favouring the former, both at post-intervention (T1) and follow-up (T2). This contrasts with the differences in adjusted means among the three groups in the initial analysis, which were all non-significant at T1 and T2. In addition, levels of loneliness among participants in the ‘high fidelity’ MFL group decreased significantly more over time than among participants in the UP group. Illustrating the implications of these findings, positive associations between perceived relatedness to others and PA-related outcomes have been found previously within group exercise settings [46]. Furthermore, higher levels of perceived relatedness have been associated with greater perceived feelings of autonomy and competence and greater wellbeing among physically active university students [28]. At the same time, emotional loneliness has been associated with all-cause mortality in older adults living alone and functional status was identified as one potential explanatory pathway [47]. Therefore, the findings regarding perceived relatedness and loneliness among participants in the ‘high fidelity’ intervention group are suggestive of the potential of the intervention, when delivered as intended, to improve psychosocial health in inactive adults aged 50 years and over.

The evidence regarding the potential of PA interventions to address increasingly critical social issues such as social isolation and loneliness in older adults is slowly growing. For example, participants in the intervention group of the Music for Movement and Health trial for community dwelling older adults scored consistently better in all psychosocial measures (social isolation, loneliness, quality of life and mood) compared to the control group post-intervention [48]. Likewise, a scoping review indicated that social activity interventions, including physical activities, can contribute to alleviate feelings of social isolation and loneliness among older adults. Importantly, though, the authors concluded that for this to happen interventions need to be tailored to the participants’ circumstances [49]. Furthermore, in a recent longitudinal study, regular participation in moderate-intensity physical activities was associated with a lower likelihood of experiencing loneliness in the future in middle-aged and older adults. However, changes in PA were not associated with variations in an individual’s typical level of loneliness [43]. Altogether, the findings from the current study and the extant literature suggest that the nature of the physical activities performed and the context (e.g., focus of the PA programmes) in which they are performed are key to understanding the potential of PA to address social isolation and loneliness in middle-aged and older adults. In this regard, programmes that combine physical and social activity appear particularly promising [50].

A similar pattern to the one observed for relatedness to others and loneliness in the analysis accounting for implementation fidelity was evident for attitudes toward PA, perceived behavioural control and intention to participate in PA. The impact of health behaviour interventions is dependent on implementation factors. Lack of effectiveness may not be a result of intervention design but instead of a failure to effectively implement the intervention [9,51]. Yet few older adult PA intervention studies seek to relate quality of implementation of an intervention to the health impact for participants; and when they do so, participant adherence (e.g., attendance) is the implementation indicator most frequently used [9]. Notably, as results from this study illustrate, implementation fidelity (i.e., extent to which an intervention is delivered as intended) is a key implementation indicator that needs to be routinely assessed if the effects of PA interventions in real world settings are to be fully understood [2]. In addition, as shown in this study, this can be accomplished in relatively simple ways while maintaining a healthy balance between pragmatism and methodological rigour, which is often a necessity in real-world research [14].

In line with previous intervention work with populations of similar characteristics to the participants in this study [20], the magnitude of the observed differences between groups in psychosocial outcomes was relatively modest. Furthermore, similar to previous studies reported in the review by Baker et al. [20], participants in this study reported relatively low levels of negative outcomes, such as loneliness, and relatively high levels of positive outcomes, such as perceived health, and relatedness to others. Relatively limited room for improvement and variability in the data, therefore, may have reduced the possibility to effect and detect change in study outcomes. Furthermore, lack of statistical power, particularly in analyses accounting for implementation fidelity, may have contributed to the latter circumstance. Another potential limitation of this study concerns the self-reported nature of implementation fidelity data. Although the ‘real world’ nature and large scale of the intervention, coupled with resource limitations, prevented us from observing and coding systematically a sufficient number of sessions, ongoing communication between the PA instructors and one research team member contributed to build trust among instructors and the research team while avoiding issues of reactivity that may arise from direct observation of sessions for coding purposes [37]. Further to this, outcomes in this study were self-reported, which may have introduced biases, such as social desirability bias. To enhance the accuracy and reliability of the data, we used several strategies. These included providing clear instructions, ensuring that questions were straightforward and easy to understand, and emphasising the confidentiality of the responses. Lastly, the total study period comprised the time from baseline to 6-month follow-up. This falls short of the minimum 6-month follow-up period post programme recommended in widely used planning and evaluation frameworks, such as RE-AIM [52]. Related to this, the different duration of some of the LSP PA programmes included in the MFL trial, which resulted in slightly different follow-up measurement periods, is another limitation of this study.

While psychosocial health and wellbeing outcomes may be difficult to change through PA interventions, particularly when these are not long-term and intensive enough, changes in the psychological determinants/mediators of health related behaviours, such as PA, can be expected as an outcome of well-designed interventions, leading eventually to changes in the behaviour itself [53]. In line with this observation, another systematic review found that the magnitude of the changes following PA interventions for older adults was larger for self-efficacy (considered a key psychological determinant/mediator of PA behaviour) than for PA [54]. Altogether, the group differences in psychological determinants/mediators of behaviour observed in this study may help explain differences in energy expenditure, body composition, and physical function outcomes reported elsewhere and favouring particularly the MFL intervention group [16]. Elements of program implementation incorporated in the MFL intervention, such as face-to-face delivery, supplemented with additional materials; and behaviour change techniques also delivered to participants as part of the intervention (e.g., instruction on how to perform PA, graded tasks, information about health consequences, autonomy and social support, goal setting and self-monitoring, action and coping planning), are useful to facilitate PA in adults through relevant mediators [2,55–57].

From the point of view of potential for adoption and scalability of the MFL intervention, the PA instructors are supported by peer mentors to deliver, and sustain, a behaviour change augmentation of existing group-based PA programs in a community setting [12]. While the model has also its limitations, determined to a large extent, as illustrated partially in this study, by the degree of ‘buy in’ from professional instructors and availability of suitable peer mentors, it offers a viable alternative to more intensive and resource demanding one-on-one counselling models of PA behaviour change used in other interventions. In addition, MFL is a behavioural augmentation of existing PA programs embedded in a delivery system of government funded LSPs, which increases its potential for adoption and scalability [8]. In this regard, the findings from this study add to the growing evidence about the feasibility and effectiveness of interventions based on augmenting or enriching collaboratively existing public PA programs for middle-aged and older adults [58].

## Conclusions

Despite a few unexpected findings, the pattern of results is reflective of the promise of MFL regarding its potential to impact positively the psychosocial health of inactive adults aged 50 years plus and change key theory-based determinants of PA behaviour in intended ways. MFL is a novel, pragmatic community-based PA programme for inactive adults aged 50 years and over designed with adoption and scalability in mind. The findings illustrate potential unintended consequences of existing community-based PA programs catering to adults and older adults and the importance of assessing implementation fidelity to fully understand the effects of PA interventions.

## Supporting information

S1 Table Details of covariate measurement and responses. (DOCX)

S2 Table Unadjusted values of outcome variables across the study groups at baseline (T0), time 1 (T1) and time 2 (T2) (mean (SD); N). (DOCX)

S3 File Consort checklist for reporting a randomised trial. (DOCX)
