# Supplementary material for: Psychosocial effects of a behavioural augmentation of existing public physical activity programs for middle-aged and older adults in Ireland
Source: PLoS One. 2025 Mar 4;20(3):e0318911. doi: 10.1371/journal.pone.0318911 (PMC11878938; doi:10.1371/journal.pone.0318911)
Supplement: S2 Table — (DOCX) [file pone.0318911.s002.docx]

S2 Table. Unadjusted values of outcome variables across the study groups at baseline (T0), time 1 (T1) and time 2 (T2) (mean (SD); N)

|  | **Time Point 0 – Study Group** | | | **Time Point 1 – Study Group** | | | **Time Point 2 – Study Group** | | |
| --- | --- | --- | --- | --- | --- | --- | --- | --- | --- |
|  | **MFL** | **UP** | **CON** | **MFL** | **UP** | **CON** | **MFL** | **UP** | **CON** |
| Wellbeing  *Score, 7-35* | 24.65 (4.47);  181 | 25.08 (4.48);  254 | 25.66 (4.63);  134 | 25.30 (4.26);  135 | 25.31 (4.30);  166 | 26.00 (4.04);  93 | 25.44 (4.04);  120 | 25.37 (4.1);  151 | 25.37 (4.09);  98 |
| Health T  *Score, 1--100* | 71.44 (15.6);  185 | 72.77 (17.3);  262 | 76.36 (17.0);  140 | 76.50; (15.25);  133 | 78.91 (14.67);  161 | 81.29 (13.30);  89 | 76.55 (17.4);  116 | 77.45 (13.9);  143 | 78.66 (16.62);  96 |
| Relatedness  *Score, 1--6* | 4.53 (1.40);  168 | 4.59 (1.22);  252 | 4.57 (1.22);  130 | 4.83 (1.03);  135 | 4.49 (1.08);  168 | 4.50 (1.14);  93 | 4.70 (1.12);  119 | 4.51 (1.04);  150 | 4.43 (1.18);  98 |
| Loneliness  *Score, 1--3* | 1.49 (0.43);  181 | 1.49 (0.46);  255 | 1.49 (0.45);  138 | 1.49 (0.47);  135 | 1.48 (0.45);  170 | 1.42 (0.47);  93 | 1.42 (0.42);  120 | 1.45 (0.47);  151 | 1.45 (0.46);  98 |
| Dec Bal  *Score, -12--12* | 1.79 (1.06);  180 | 1.96 (0.98);  248 | 1.95 (1.00);  132 | 1.72 (1.07);  135 | 1.86 (0.95);  164 | 1.96 (0.96);  93 | 1.68 (1.05);  120 | 1.89 (0.98);  151 | 1.78 (0.93);  98 |
| Barriers SE  *Score, 1--5* | 2.69 (0.78);  181 | 2.86; (0.81)  252 | 2.91; (0.80)  138 | 2.84 (0.74);  135 | 2.67 (0.79);  164 | 2.94 (0.74);  93 | 2.82 (0.80);  120 | 2.72 (0.74);  151 | 2.97 (0.72);  98 |
| Attitude  *Score, 1--4* | 3.38 (0.42);  181 | 3.37 (0.42);  261 | 3.31 (0.46);  137 | 3.31 (0.42);  135 | 3.25 (0.48);  168 | 3.33 (0.41);  93 | 3.31 (0.45);  120 | 3.27 (0.44);  150 | 3.36 (0.44);  98 |
| Subj norm  *Score, 1--4* | 2.83 (0.57);  167 | 2.78 (0.57);  237 | 2.60 (14.2);  130 | 2.83 (0.51);  135 | 2.71 (0.59);  163 | 2.74 (0.48);  93 | 2.84 (0.49);  119 | 2.74 (0.48);  149 | 2.73 (0.51);  98 |
| Beh control  *Score, 1--4* | 3.35 (0.50);  180 | 3.30 (0.49);  260 | 3.35 (0.48);  140 | 3.24 (0.47);  135 | 3.23 (0.53);  167 | 3.27 (0.49);  93 | 3.19 (0.45);  120 | 3.21 (0.43);  151 | 3.32 (0.52);  98 |
| Intention  *Score, 1--4* | 3.34 (0.55);  185 | 3.29 (0.50);  261 | 3.22 (0.52);  141 | 3.20 (0.45);  125 | 3.10 (0.61);  169 | 3.12 (0.48);  93 | 3.13 (0.51);  120 | 3.04 (0.54);  151 | 3.18 (0.53);  98 |

*Note.* MFL = Move for Life Intervention Group, UP = Usual Provision, CON = Control; Health T = health thermometer, Dec Bal = decisional balance, Barriers SE = barriers self-efficacy, Subj norm = subjective norms, Beh control = perceived behavioural control.
